# Supplementary material for: Identification of Freezing-Responsive microRNAs and Their Targets in Chinese Jujube by Small RNA and Degradome Sequencing
Source: Int J Mol Sci. 2026 Sep 4;27(17):7912. doi: 10.3390/ijms27177912 (PMC13566890; doi:10.3390/ijms27177912)
Supplement: Supplementary file 1 [file ijms-27-07912-s001.zip › Supplementary Material S2/novel_miR_182.pdf]

Provisional ID	:	novel_miR_182
Score total	:	900.9
Score for star read(s)	:	3.9
Score for read counts	:	894.4
Score for mfe	:	1
Score for randfold	:	1.6
Score for cons. seed	:	
Total read count	:	1766
Mature read count	:	1748
Loop read count	:	0
Star read count	:	18

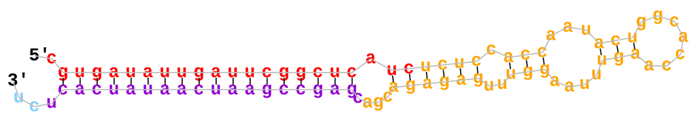

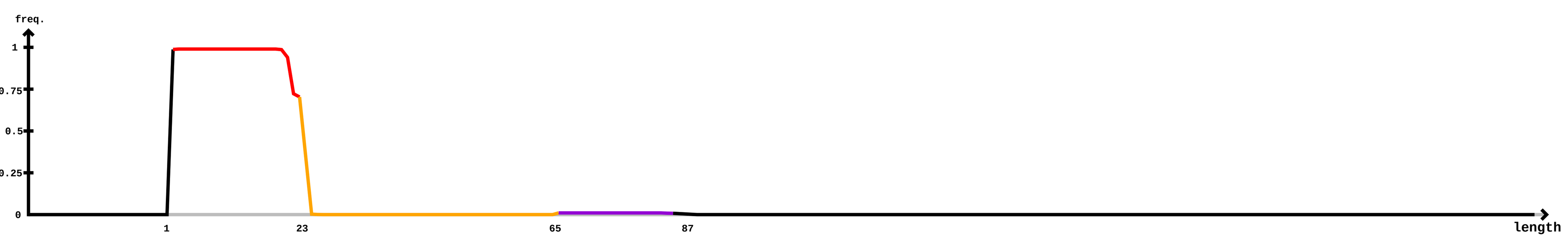

Mature Star

```
5'- cauucugcagaaguagacauggcgugauauugauucggcucaucucuccaccaauacuggcaccaaguuuaagguuugagagacgacgagccgaaucaauaucacucuuguaugcuucuucgcauauuaucaagguuuuuccuguuuuuugaguuccaauauuagccaagaagcaucucugcaggcaacucaugcauuaagcuacauagaagcuuguuuuggcucuccaucccccucuuguaaagucuuc -3' obs
    cauucugcagaaguagacauggcgugauauugauucggcucaucucuccaccaauacuggcaccaaguuuaagguuugagagacgacgagccgaaucaauaucacucuuguaugcuucuucgcauauuaucaagguuuuuccuguuuuuugaguuccaauauuagccaagaagcaucucugcaggcaacucaugcauuaagcuacauagaagcuuguuuuggcucuccaucccccucuuguaaagucuuc exp
    ....((((((.........((.((((((((((((((((((.((((((.(((...(((.......)))....)))..))))))....))))))))))))))))))).)).((((((......))))))......((.....))(((((((((.(((........))))))))))))..)))))))(((.....)))..((((((.......))))))....((((...((.........))...))))...
```

	reads	mm	sample
......................cgugauauugauucggcu	1	0	LT-1
......................cgugauauugauucggcu	5	0	LT-3
......................cgugCuauugauucggcuc	1	1	LCK-3
......................cgugauauugauucggcuc	33	0	LCK-3
......................cgugauauugauucggcuc	13	0	LT-3
......................cgugauauugauucggcuU	1	1	LT-3
......................cgugauauugauucggcuc	7	0	LT-1
......................cgugauauugauucggcuc	21	0	LT-2
......................cgugauauugauucggcuA	6	1	LCK-3
......................cgugauauugauucggcuca	71	0	LT-2
......................cgugauauugauucggcuca	3	0	JT-3
......................cgugauauugauucggcuca	48	0	LCK-1
......................cgugauCuugauucggcuca	1	1	LCK-1
......................cgugauauugauucggcuca	3	0	JCK-3
......................cgugauauugauucggcuca	2	0	JCK-1
......................cgugauauugauucggcucU	3	1	LT-2
......................cgugauauugauucggcuca	103	0	LCK-3
......................cgugauCuugauucggcuca	1	1	LCK-2
......................cgugauauugauucggcuca	60	0	LCK-2
......................cgugauauugauucggcuca	1	0	JT-2
......................cgugauauugauucggcuca	23	0	LT-1
......................cgugauCuugauucggcuca	1	1	LT-3
......................cAugauauugauucggcuca	1	1	LT-2
......................cgugauauugauucggcuca	9	0	JCK-2
......................cgugauauugauucggcuca	52	0	LT-3
......................cgugauauugauucgAcuca	1	1	LCK-3
......................cgugauauugauucggcucau	8	0	LT-1
......................cgugauauugauucggcucau	14	0	LCK-3
......................cgugauauugauucggcucau	1	0	LT-3
......................cgugauauugauucggcucau	9	0	LT-2
......................cgugauCuugauucggcucau	1	1	LT-3
......................cgugauauugauucggcucaA	1	1	LT-3
......................cgugauauugauucggcucauU	5	1	JCK-3

Mature / Star

cauucugcagaaguagacaugg**cgugauauugauucggcucauc**ucuccaccaauacuggcaccaaguuuaagguuugagagacga**cgagccgaaucaauaucacu**cuuguaugcuucuucgcauauuaucaagguuuuuccuguuuuuugaguuccaauauuagccaagaagcaucucugcaggcaacucaugcauuaagcuacauagaagcuuguuuuggcucuccaucccccucuuguaaagucuuc			
......................cgugauauugauucggcucauc	122	0	LT-2
......................cgugauauugauucggcucauc	99	0	JCK-2
......................cgugauauugauucggcucauU	5	1	LT-3
......................cgugauauugCuucggcucauc	1	1	JCK-2
......................cgugauauugauucggcucauc	31	0	LCK-1
......................cgugauauugauucggcucauU	13	1	LT-2
......................cgugauauugauucggcucauc	31	0	LCK-2
......................cgugauauugauucggcucauc	237	0	LT-1
......................cgugauauugauucggcucauc	137	0	LCK-3
......................cgugauauugauucggcucCuc	1	1	LT-2
......................cgugCuauugauucggcucauc	1	1	LT-3
......................cgugauauugauucggcucauU	1	1	JCK-2
......................cgGgauauugauucggcucauc	1	1	LT-2
......................cgugauauugauucggcucauc	93	0	JCK-3
......................cgugauauugauucggcucauc	248	0	LT-3
......................cgugauauugauucggcucauc	51	0	JCK-1
......................cguUauauugauucggcucauc	1	1	LT-2
......................cgugauauugauucggcucauU	1	1	JCK-1
......................cgugauauugauucggcucauU	3	1	LT-1
......................cgugCuauugauucggcucauc	2	1	JCK-2
......................cgugauauugCuucggcucauc	1	1	JCK-1
......................cNugauauugauucggcucauc	1	1	JCK-1
......................cgugauauugauucggcucauU	9	1	LCK-3
......................Agugauauugauucggcucauc	1	1	LT-1
......................cgugauauugCuucggcucauc	1	1	LT-1
......................cguAauauugauucggcucauc	1	1	JCK-3
......................cgugCuauugauucggcucauc	1	1	LCK-3
......................cgugauauugauucggcCcauc	1	1	LT-1
......................cgugauauugauucggcucaucu	30	0	LT-1
......................cgugauauugauucggcucauUu	2	1	LT-1
......................cgugauauugauucggcucauUu	9	1	LCK-3
......................cgugauauugauucggcucaucu	17	0	LT-2
......................cgugauauugauucggcucaucA	1	1	LCK-3
......................cgugauauugauucggcucaucu	3	0	JCK-2
......................cgugauauugauucggcucauUu	6	1	LT-2
......................cgugauauugauucggcucaucA	2	1	LT-1
......................cgugauauugCuucggcucaucu	1	1	LT-1
......................cgugauauugauucggcucaucu	4	0	JCK-3
......................cgugauauugauucggcucauUu	1	1	LT-3
......................cgugauauugauucggcucaucu	15	0	LCK-3
......................cAugauauugauucggcucaucu	1	1	LT-2
......................cgugauauugauucggcucauAu	1	1	LT-2
......................cgugauauugauucggcucaucu	34	0	LT-3
......................cgugauauugauucggcucaucA	3	1	LT-2
......................cgugauauugauucggcucaucu	1	0	JCK-1
......................cgugauauugauucggcucaucA	3	1	LT-3
......................cgugauauugauucggcucaucuU	1	1	LT-2
......................cgugauauugauucggcucaucuU	2	1	LT-3
......................cgugauauugauucggcucaucuU	1	1	JCK-2
......................cgugauauugauucggcucaucuUu	1	1	LCK-3
.......................gugauauugauucggcuca	1	0	LT-2
.......................gugauauugauucggcucauc	1	0	LT-1
.......................gugauauugauucggcucauc	2	0	LT-3
....cgagccgaaucaauauca	1	0	LT-2
....cgagccgaaucaauaucU	2	1	LCK-3
....cgagccgaaucaauaucac	1	0	LT-1
....cgagccgaaucaauaucacu	5	0	LT-2
....cgagccgaaucaauaucacu	2	0	LT-3
....cgagccgaaucaauaucacu	6	0	LT-1
....cgagccgaaucaauaucacuU	1	1	LT-1
